# Efficacy of anti-PD-1 antibodies in NSCLC patients with an *EGFR* mutation and high PD-L1 expression

**DOI:** 10.1007/s00432-020-03329-0

**Published:** 2020-07-23

**Authors:** Ken Masuda, Hidehito Horinouchi, Midori Tanaka, Ryoko Higashiyama, Yuki Shinno, Jun Sato, Yuji Matsumoto, Yusuke Okuma, Tatsuya Yoshida, Yasushi Goto, Noboru Yamamoto, Yuichiro Ohe

**Affiliations:** 1 Department of Thoracic Oncology, National Cancer Center Hospital, 5-1-1 Tsukiji, Chuo-ku, Tokyo, 104-0045 Japan; 2 Department of Experimental Therapeutics, National Cancer Center Hospital, Tokyo, Japan

**Keywords:** Non-small cell lung cancer, Programmed death-ligand-1, Epidermal growth factor receptor, Immune checkpoint inhibitor

## Abstract

**Introduction:**

Several studies have demonstrated that non-small cell lung cancer patients (NSCLCs) harboring epidermal growth factor receptor (*EGFR*) mutations have poor clinical outcomes in response to treatment with programmed death-1 (PD-1) inhibitors. However, it remains unclear whether EGFR-mutated NSCLCs with a high programmed death-ligand-1 (PD-L1) expression (tumor proportion score ≥ 50%) respond to PD-1 inhibitors.

**Methods:**

We retrospectively investigated the NSCLCs who had received PD-1 inhibitors between January 2016 and December 2018 to assess the efficacy of PD-1 inhibitors in patients with an *EGFR* mutation and high PD-L1 expression.

**Results:**

There were 153 patients with a high PD-L1 expression level, and the median progression-free survival (mPFS) was 5.3 months [95% confidence interval (CI) 1.3–12.4 months] in the patients with *EGFR* mutations (*n* = 17) and 8.3 months (95% CI 6.0–11.7 months) in those with wild-type *EGFR* (*n* = 136; hazard ratio (HR) 1.62; 95% CI 0.83–2.87). Among the 110 patients in the low PD-L1 expression group, the mPFS was 1.6 months (95% CI 1.3–5.9 months) in the patients with *EGFR* mutations (*n* = 18) and 3.8 months (95% CI 2.5–5.9 months) in those with wild-type *EGFR* (*n* = 92; HR 2.59; 95% CI 1.48–4.31). The HR for PFS in the group with *EGFR* mutations and high PD-L1 expression was 0.97 (95% CI 0.56–1.59) compared to the group with wild-type *EGFR* and low PD-L1 expression.

**Conclusions:**

PD-1 inhibitors can serve as one of the treatment options for NSCLCs with an *EGFR* mutation and high PD-L1 expression.

## Introduction

Immune checkpoint inhibitors (ICIs), particularly inhibitors of the programmed death-1 (PD-1) axis, have revolutionized the treatment of non-small cell lung cancer (NSCLC). Treatment with ICIs has been shown to result in a significant tumor response and overall survival (OS) benefit in advanced NSCLC (Borghaei et al. 2015; Brahmer et al. 2015; Mok et al. 2019; Reck et al. 2016). Programmed death-ligand-1 (PD-L1) expression in tumor cells is associated with improved clinical outcomes of PD-1 pathway blockade in NSCLC patients (Garon et al. 2015; Herbst et al. 2014). Pembrolizumab monotherapy has become a standard first-line treatment for advanced NSCLC in patients with a PD-L1 tumor proportion score (TPS) of at least 50%, based on the results of the KEYNOTE-024 phase III trial (Reck et al. 2016). Several studies have also shown a relationship between high PD-L1 expression and a higher objective response rate (ORR) and better survival in NSCLC patients treated with PD-1 inhibitors, including nivolumab and pembrolizumab (Aguiar et al. 2017). However, most clinical studies have excluded specific patients, for example, patients with epidermal growth factor receptor (*EGFR*) mutations.

Several studies have reported disappointing clinical outcomes with lower response rates and shorter survival in patients with EGFR-mutated NSCLC treated with PD-1 inhibitors than in patients with EGFR-wild NSCLC (Bylicki et al. 2017; Gainor et al. 2016; Lee et al. 2018; Santambrogio and Rammensee 2019). *EGFR* tyrosine kinase inhibitors (EGFR-TKIs) are standard first-line treatment for EGFR-mutated NSCLC. Lisberg et al. reported a phase II trial of pembrolizumab in TKI-naive patients with advanced EGFR-mutated, PD-L1-positive NSCLC and concluded that pembrolizumab is not appropriate as a first-line treatment for EGFR-mutated NSCLC before EGFR-TKI therapy (Lisberg et al. 2018). However, it remained unclear whether EGFR-mutated NSCLC with high PD-L1 expression (TPS ≥ 50%) responds to ICIs, because the sample size in their trial was too small. We retrospectively investigated the relationship between PD-L1 expression and the efficacy of PD-1 inhibitors in NSCLC patients to assess the efficacy of PD-1 inhibitors in patients with an *EGFR* mutation and high PD-L1 expression.

## Materials and methods

### Study design

This study was a retrospective, single-center, observational study conducted at the National Cancer Center Hospital in Japan. The study was approved by the Institutional Review Board of the National Cancer Center Hospital (No. 2015-355).

### Subjects

Patients with advanced NSCLC who had been treated with an anti-PD-1 antibody between March 2017 and December 2018 at the National Cancer Center Hospital in Japan were identified from the database. Patients with no PD-L1 expression data were excluded. We reviewed the medical records and abstracted the following patient characteristics: age, gender, Eastern Cooperative Oncology Group Performance Status (ECOG-PS), histology, disease status, *EGFR* mutation status, details of treatment, and survival. PD-L1 expression was evaluated using the PD-L1 22C3 pharmDx (Dako, Carpinteria, CA, USA) and *EGFR* mutations were identified using the Cobas® EGFR Mutation Test v2 (Cobas; Roche Diagnostics, Basel, Switzerland). The patients who were adopted as subjects of our study were divided into four groups according to PD-L1 expression level and EGFR mutation status. In our study, low PD-L1 expression was defined as the presence of < 50% positive-staining tumor cells, whereas ≥ 50% positive staining was considered high PD-L1 expression. The efficacy of treatment with the PD-1 inhibitors in the four groups was assessed by evaluating progression-free survival (PFS).

### Treatment and assessment

In the safety analysis, we evaluated adverse events associated with ICIs or EGFR-TKIs according to the National Cancer Institute Common Terminology Criteria for Adverse Events, version 4.03. Objective tumor response in patients with target lesions was evaluated based on the Response Evaluation Criteria in Solid Tumors version 1.1 and assessment by computed tomography every 6–8 weeks after the start of treatment.

### Statistical analysis

Differences between groups were analyzed using Fisher’s exact test for categorical variables. PFS was defined as the time between the start of PD-1 inhibitor treatment and progression or death from any cause; PFS was censored at a date when the patient was confirmed to be progression free. Patients whose treatment was discontinued due to toxicity in the absence of disease progression were censored at the start of the next treatment. Overall survival (OS) was measured until death or censored at the latest follow-up examination of surviving patients. Survival rates were estimated by the Kaplan–Meier method and compared using the log-rank test. All statistical analyses were performed using the JMP version 14.0 software program (SAS Institute, Cary, NC, USA). All *P* values were two sided, and *p* < 0.05 was considered evidence of a statistically significant difference.

## Results

### Patient characteristics

**Fig. 1:**
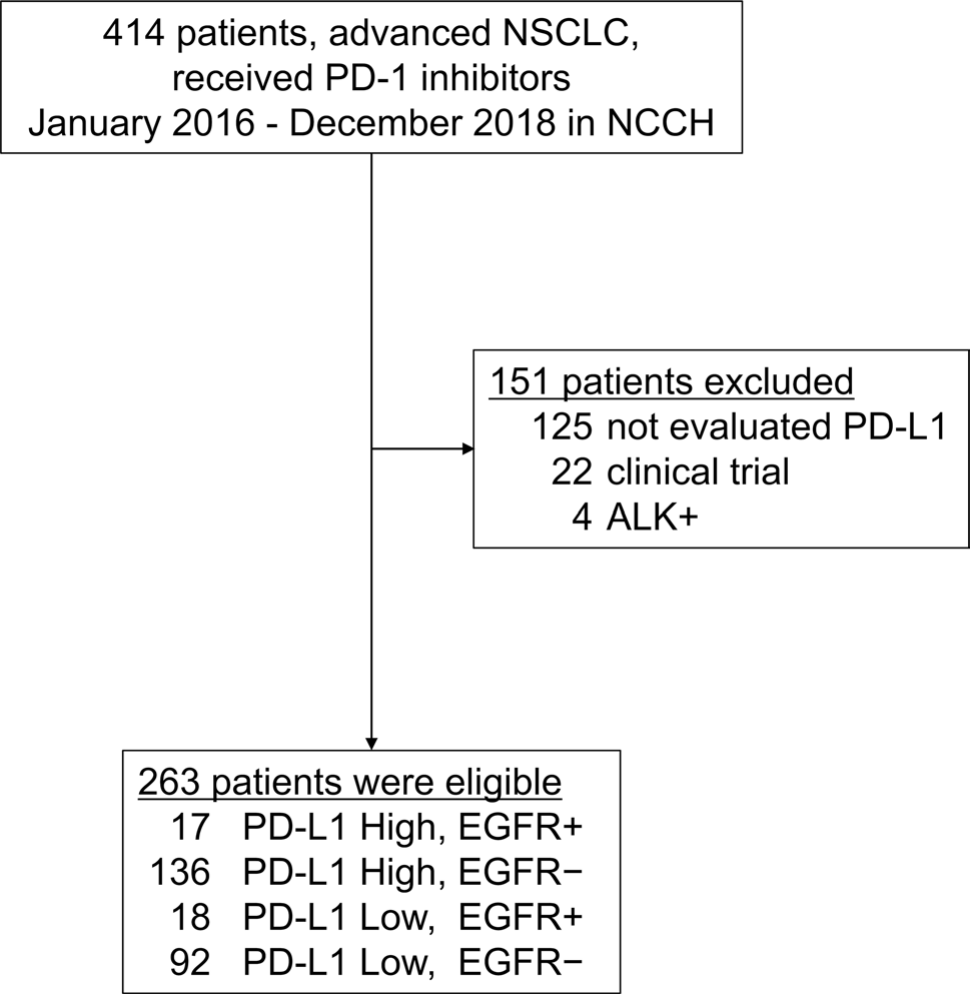
Patient selection. Of the 414 non-small cell lung cancer (NSCLC) patients treated with nivolumab or pembrolizumab at the National Cancer Center Hospital in Japan between March 2017 and December 2018, the 263 patients were adopted as the subjects of this study and divided into 4 groups based on their programmed death-ligand-1 (PD-L1) expression level and *EGFR* mutation status. The reasons for excluding 151 patients were absence of PD-L1 data (*n* = 125), participation in a clinical trial of pembrolizumab or nivolumab (*n* = 22), and NSCLC with *ALK* rearrangement (*n* = 4)

In this study, the 414 NSCLC patients treated with nivolumab or pembrolizumab at the National Cancer Center Hospital between March 2017 and December 2018 were identified as candidates for inclusion, and 263 of them were ultimately adopted as subjects of our study. We excluded 151 patients for the following reasons: absence of PD-L1 data (*n* = 125), participation in a clinical trial of pembrolizumab or nivolumab (*n* = 22), and NSCLC with *ALK* rearrangement (*n* = 4) (Fig. 1). The median age of the subjects was 62 years (range 33–87 years). High PD-L1 expression was found in 153 patients (58.2%). Thirty-five (7.5%) patients had *EGFR* mutations, and 29 (82.9%) of these 35 patients had an exon 19 deletion or exon 21 L858R mutation (Table 1).

**Table 1:** Patient characteristics

	All patients *N*	PD-L1 high EGFR + *N*	PD-L1 high EGFR − *N*	PD-L1 low EGFR + *N*	PD-L1 low EGFR − *N*
Total *N*	263	17	136	18	92
Median age, years (range)	62 (33–87)	62 (47–85)	62 (33–87)	64.5 (37–83)	62 (33–83)
Sex					
Female	83	7	36	15	25
Male	180	10	100	3	67
ECOG-PS					
0, 1	236	14	125	16	81
2	27	3	11	2	11
Smoking history					
Never smoker	53	7	21	12	13
Smoker	210	10	115	6	79
Histologic classification					
Adenocarcinoma	203	16	107	18	62
Squamous	52	0	24	0	28
Others	8	1	5	0	2
Disease status					
Stage IV	140	9	75	12	44
Stage III	53	3	30	2	18
Recurrence	70	5	31	4	30
EGFR mutation status					
Ex19del	21	8	0	13	0
L858R	8	6	0	2	0
Others	6	3	0	3	0
Negative	0	0	136	0	92
ICIs status					
Pembrolizumab	141	11	105	4	21
Nivolumab	122	6	31	14	71
Line of ICI					
First-line	92	2	85	0	5
Second-line	111	3	42	2	64
Third-line or more	60	12	9	16	23

*ECOG-PS* Eastern Cooperative Oncology Group Performance Status, *EGFR* epidermal growth factor receptor, *ICI* immune checkpoint inhibitors, *PD-L1* programmed death-ligand 1

### Efficacy

**Fig. 2:**
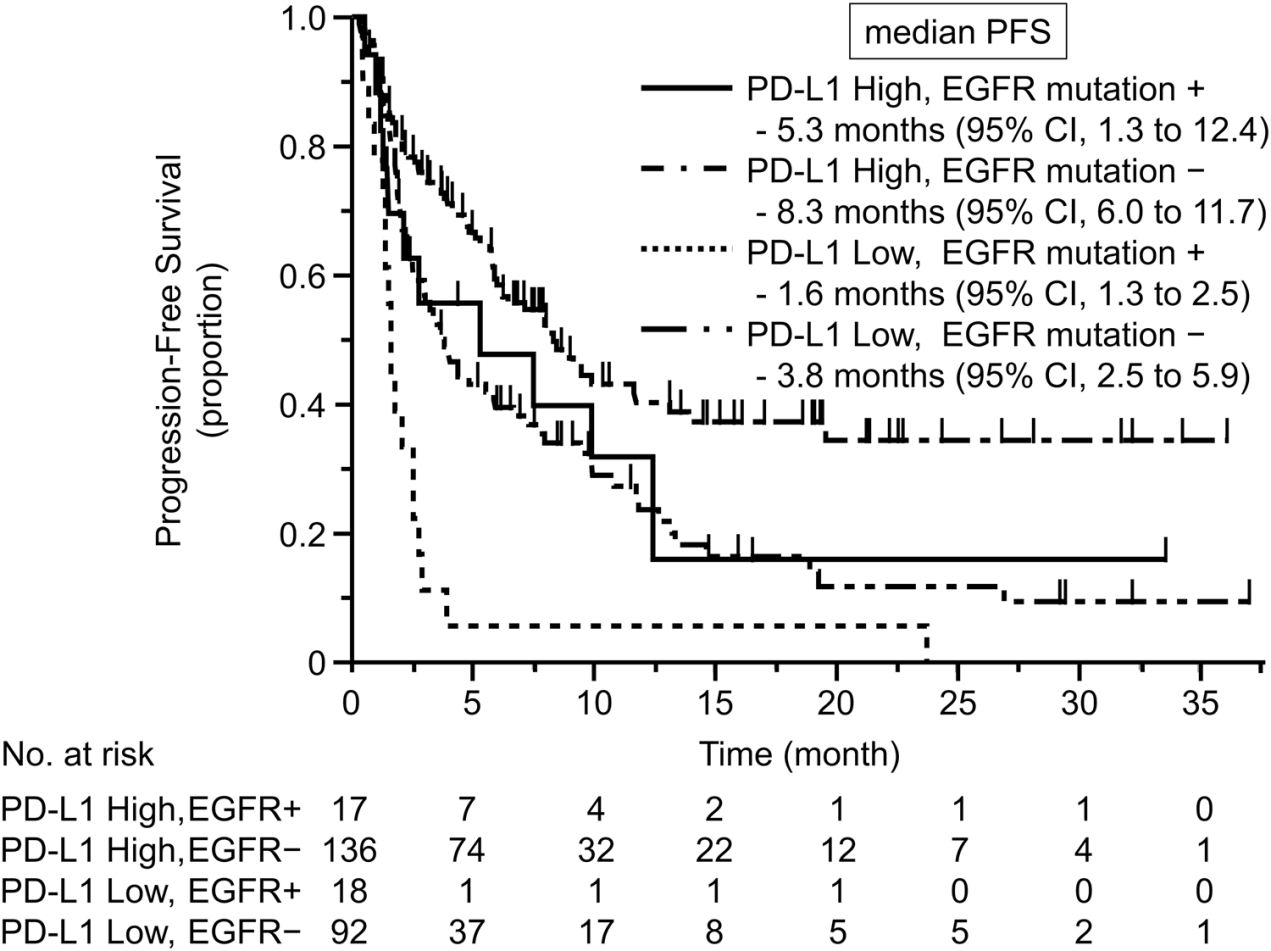
Kaplan–Meier curve for progression-free survival (PFS) according to PD-L1 expression and *EGFR* mutation status. In the high PD-L1 expression group, median PFS was 5.3 months (95% CI 1.3–12.4 months) in the *EGFR* mutation subgroup and 8.3 months (95% CI 6.0–11.7 months) in the wild-type *EGFR* subgroup. In the low PD-L1 expression group, median PFS was 1.6 months (95% CI 1.3–2.5 months) in the *EGFR* mutation subgroup and 3.8 months (95% CI 2.5–5.9 months) in the wild-type *EGFR* subgroup

The median follow-up time was 11.3 months [95% confidence interval (CI) 9.0–14.7 months]. Table 2 summarizes the efficacy of the PD-1 inhibitors. Kaplan–Meier curves for PFS according to PD-L1 expression level and *EGFR* mutation status are shown in Fig. 2. In the high PD-L1 expression group, the ORR was 29.4% (95% CI 1.3–53.1%) in the *EGFR* mutation subgroup (*n* = 17) and 43.4% (95% CI 35.4–51.8%) in the wild-type *EGFR* subgroup (*n* = 136). Median PFS was 5.3 months (95% CI 1.3–12.4 months) in the *EGFR* mutation subgroup and 8.3 months (95% CI 6.0–11.7 months) in the wild-type *EGFR* subgroup [hazard ratio (HR) 1.62; 95% CI 0.83–2.87; *p* = 0.125]. In the low PD-L1 expression group, the ORR was 0% in the *EGFR* mutation subgroup (*n* = 18) and 16.3% (95% CI 10.1–25.2%) in the wild-type *EGFR* subgroup (*n* = 92). Median PFS was 1.6 months (95% CI 1.3–2.5 months) in the *EGFR* mutation subgroup and 3.8 months (95% CI 2.5–5.9 months) in the wild-type *EGFR* subgroup (HR 0.39; 95% CI 0.23–0.66; *p* < 0.001). The PFS of the group with *EGFR* mutations and high PD-L1 expression was similar to the PFS in the group with wild-type *EFGR* and low PD-L1 expression (HR 0.97; 95% CI 0.56–1.59; *p* = 0.909). In the *EGFR* mutation group, median OS was 26.4 months (95% CI, 6.7 to not evaluated) in the high PD-L1 expression subgroup and 12.7 months (95% CI 2.6 to not evaluated) in the low PD-L1 expression subgroup. In the wild-type *EGFR* group, median OS was 36.2 months (95% CI 21.0–36.2 months) in the high PD-L1 expression subgroup and 13.0 months (95% CI 9.9–29.7 months) in the low PD-L1 expression subgroup. Regarding the patterns of progression after PD-1 inhibitors, there was no significant difference between the *EGFR* mutation group and the wild-type *EGFR* group.

**Table 2:** Summary of the efficacy of PD-1 inhibitors

	ORR (%) 95% CI	mPFS (month) 95% CI	HR of mPFS 95% CI
PD-L1 high EGFR −*N* = 136	43.4 35.4–51.8	8.3 6.0–11.7	0.56 0.40–0.78
PD-L1 high EGFR + *N* = 17	29.4 1.3–53.1	5.3 1.3–12.4	0.97 0.56–1.59
PD-L1 low EGFR −*N* = 92	16.3 10.1–25.2	3.8 2.5 to 5.9	Reference
PD-L1 low EGFR −*N* = 18	0	1.6 1.3–2.5	2.59 1.48–4.31

*CI* confidence interval, *EGFR* epidermal growth factor receptor, *HR* hazard ratio, *mPFS* median progression-free survival, *ORR* objective response rate, *PD-L1* programmed death-ligand 1

### Toxicity

An immune-related adverse event (irAE) developed in 5 (29.4%) of the 17 patients with EGFR-mutated NSCLC and high PD-L1 expression. The most frequent adverse events in this study were diarrhea (*n* = 2) and hypothyroidism (*n* = 2). Grade 3 alanine and aspartate aminotransferase elevation was observed in one patient. Grade 4 small intestinal perforation occurred in one patient treated with nivolumab, and nivolumab was discontinued; however, PD-1 inhibitor therapy was continued after the irAE in the other patients. There were no grade 5 adverse events related to the PD-1 inhibitors.

## Discussion

The results of our study showed that PD-L1 expression was associated with the efficacy of PD-1 inhibitors in patients with *EGFR* mutations. The ORR and median PFS in the high PD-L1 expression group were 29.4% (95% CI 1.3–53.1%) and 5.3 months (95% CI 1.3–12.4 months), respectively, compared with 0% and 1.6 months (95% CI 1.3–2.5 months), respectively, in the low PD-L1 expression group. In the group of patients with an EGFR mutation, the efficacy of the PD-1 inhibitors was greater in the subgroup of patients with high PD-L1 expression than in the subgroup with low PD-L1 expression. Moreover, PFS in the group with EGFR mutations and high PD-L1 expression was similar to PFS in the group with wild-type EFGR and low PD-L1 expression (HR 0.97; 95% CI 0.56–1.59; *p* = 0.909).

Berghoff et al. recently reviewed ICI treatment in patients with oncogene-addicted NSCLC (Berghoff et al. 2019). They evaluated the efficacy of ICIs in NSCLC patients with wild-type EGFR and in patients with EGFR-mutated NSCLC in five clinical trials: CheckMate 057 (Borghaei et al. 2015), KEYNOTE-010 (Herbst et al. 2016), OAK (Rittmeyer et al. 2017), POPLAR (Fehrenbacher et al. 2016), and IMpower150 (Socinski et al. 2018), and found that the survival benefits of treatment with an ICI tended to be lower in patients with EGFR mutations than in patients with wild-type EGFR. Lee et al. performed a meta-analysis study that assessed the role of ICIs as second-line therapy in advanced EGFR-mutated NSCLC (Lee et al. 2018). Their analysis of the data from three clinical trials (CheckMate 057 (Borghaei et al. 2015), KEYNOTE-010 (Herbst et al. 2016), and POPLAR (Fehrenbacher et al. 2016)) showed that ICIs did not improve OS compared with docetaxel therapy. Both meta-analyses also evaluated the results of ICI therapy in PD-L1-positive NSCLC, but there have been no reports on the efficacy of ICIs in patients with EGFR-mutated NSCLC and high PD-L1 expression. Our own data showed that PD-1 inhibitors were beneficial as second-line or later treatment of patients with EGFR-mutated NSCLC and high PD-L1 expression.

Data regarding the relative risk of toxicity with ICIs and EGFR-TKIs in NSCLC patients in several studies have revealed more severe irAEs when EGFR-TKIs were used in combination with ICIs or used after ICIs. Ahn et al. reported that a phase Ib clinical trial of concurrent durvalumab (anti-PD-L1 agent) plus osimertinib was halted due to a high rate of interstitial lung disease (Ahn et al. 2016). Schoenfeld et al. found that treatment with an ICI followed by osimertinib was associated with severe irAEs (Schoenfeld et al. 2019), but no irAEs were observed in their study when osimertinib preceded ICI therapy or when treatment with an ICI was followed by other EGFR-TKIs. A case reported by Kaira et al. showed that EGFR-TKI re-challenge immediately after nivolumab therapy may be tolerable and effective in patients with EGFR-TKI resistance (Kaira and Kagamu 2019). Whether irAEs are more severe when EGFR-TKIs are used in combination with ICIs or after ICIs remains a matter of controversy. If future investigations elucidate the mechanisms of toxicity and clinical situations in which toxicity develops, it might be possible to provide better treatment options and clinical benefits to patients with EGFR-mutated NSCLC and high PD-L1 expression.

This study had several limitations. First, this study was retrospective and conducted in a single center. The follow-up periods were not identical; however, all patients were regularly followed up every 1–2 months as outpatients, and evaluations were performed every 3–6 months for 1 year. In addition, their condition was subsequently checked every 6 months by X-ray, computed tomography (CT), magnetic resonance imaging, or positron emission tomography CT. Second, patient characteristics were not uniform across the groups, and that may have led to selection bias.

## Conclusions

In conclusion, our study showed that patients with EGFR-mutated NSCLC and higher PD-L1 expression received a greater benefit of treatment with PD-1 inhibitors in terms of ORR and PFS than patients with low PD-L1 expression did. In addition, the ORR and PFS in the group of NSCLC patients with an *EGFR* mutation and high PD-L1 expression were similar to the ORR and PFS in the group with wild-type EFGR and low PD-L1 expression. The findings in our study suggest that even in NSCLC patients with an *EGFR* mutation evaluation of PD-L1 expression can help predict the efficacy of PD-1 inhibitors, and that PD-1 inhibitors can serve as one of the treatment options for patients with an* EGFR* mutation and high PD-L1 expression.

## Data Availability

The datasets generated during the current study are not publicly available due to ethical restrictions, but are available from the corresponding author on reasonable request.
